# Thonzonium bromide inhibits progression of malignant pleural mesothelioma through regulation of ERK1/2 and p38 pathways and mitochondrial uncoupling

**DOI:** 10.1186/s12935-024-03400-7

**Published:** 2024-06-29

**Authors:** Irene Dell’Anno, Federica Morani, Simone Patergnani, Antonio Daga, Paolo Pinton, Carlotta Giorgi, Luciano Mutti, Federica Gemignani, Stefano Landi

**Affiliations:** 1 Department of Biology, University of Pisa, Pisa, Italy; 2 Department of Medical Sciences, Section of Experimental Medicine, Laboratory for Advanced Therapies (LTTA), Technopole of Ferrara, Ferrara, Italy; 3 IRCCS, Ospedale Policlinico San Martino, Genoa, Italy; 4 Sbarro Institute for Cancer Research and Molecular Medicine, Center for Biotechnology, College of Science and Technology, Temple University, Philadelphia, USA; 5 Department of Biotechnological and Applied Clinical Sciences, University of L’Aquila, L’Aquila, Italy

**Keywords:** Malignant pleural mesothelioma, Thonzonium bromide, NOD-SCID, Mitochondrial uncouplers

## Abstract

**Background:**

Malignant Pleural Mesothelioma (MPM) is a rare malignancy with a poor prognosis. Current therapies are unsatisfactory and novel cures are urgently needed. In a previous drug screening, we identified thonzonium bromide (TB) as one of the most active compounds against MPM cells. Since the biological effects of TB are poorly known, in this work we departed from some hints of previous studies and investigated several hypotheses. Moreover, we evaluated the efficacy of TB in an in vivo xenograft rodent model.

**Methods:**

In vitro assessment was made on five MPM (Mero-14, Mero-25, Ren, NCI-H28, MSTO-211H) and one SV40-immortalized mesothelial cell line (MeT-5A). We evaluated TB ability to affect proliferation, apoptosis, mitochondrial functions and metabolism, and the mevalonate pathway. In vivo assay was carried out on MPM-xenograft NOD-SCID mice (4 mg/kg delivered intraperitoneally, twice a week for 4 weeks) and the overall survival was analysed with Kaplan-Meier curves.

**Results:**

After TB treatment, we observed the suppression of ERK 1/2 phosphorylation, the increase of BAX expression and p38 phosphorylation. TB affected Ca^2+^ homeostasis in both mitochondrial and cytosolic compartments, it regulated the mitochondrial functioning, respiration, and ATP production as well as the mevalonate pathway. The in vivo study showed an increased overall survival for TB treated group *vs*. vehicle control group (*P* = 0.0076).

**Conclusions:**

Both in vitro and in vivo results confirmed the effect of TB on MPM and unravelled novel targets with translational potential.

**Supplementary Information:**

The online version contains supplementary material available at 10.1186/s12935-024-03400-7.

## Introduction

Malignant Pleural Mesothelioma (MPM) is a lethal cancer with few therapeutic options, which makes the identification of novel actionable targets and molecules for improved care urgent [1]. A possible strategy for accelerating this process could consist in repositioning known and already approved drugs for novel uses. The approach of “drug repurposing” provides interesting results and several examples are well-known, including thalidomide, zoledronic acid, celecoxib, methotrexate, and gemcitabine [2]. Following this concept, in a previous work, we carried out an in vitro screening on immortalized and patient-derived primary MPM cell lines of 1170 FDA-approved drugs. We reported cephalomannine, ouabain, emetine and thonzonium bromide (TB) as the lead compounds that were highly toxic for the malignant Mero-14, Mero-25, IST-Mes2, NCI-H28, Ren, and MSTO-211H cell lines, with TB being the most active [3]. This was the first evidence that TB had toxic activity on malignant cell lines. Indeed, TB is used as a surfactant for eardrops, and it is also employed as a preservative for its activity against fungal pathogens and parasites. TB is also known as an anthelmintic acting against *Cooperia oncophora* [4] and as an antifungal against a wide range of multidrug-resistant fungi [5]. Moreover, it was found active on methicillin-resistant *Staphylococcus aureus* strains [6] and on a broad range of bacteria [7]. However, the mechanisms underlying these effects are poorly known. Most antiparasitic agents target mitochondria [8, 9], therefore studies on the activity at this level are fully warranted. Indeed, TB was also identified, by Chan and collaborators, as a specific *Candida albicans* vacuolar “V” ATPase inhibitor [10]. This pump, through its two domains V_1_ and V_0_, functionally and structurally couples ATP hydrolysis and proton transport via a rotational mechanism of catalysis. Interestingly, TB was shown to block proton transport but not the ATP hydrolysis and thereby it was termed as “uncoupler” of the V-ATPase proton pumps. As a result, uncouplers molecules (e.g. salicylihalamide [11]) can acidify the yeast cytosol, inhibit ATP-dependent proton transport in vacuolar membrane fractions and cause pH-sensitive growth defects, characteristic of yeast cells with impaired V-ATPase function. However, the essential role of this type of ATPases is to synthesize ATP from ADP and inorganic phosphate at the expense of an electrochemical proton gradient. For this reason, they have been termed as “ATPase-ATP synthases” and their structures are highly related to those found in chloroplasts and mitochondria [12]. Translating to mammalian cells, the impairment of ATP synthase largely affects mitochondrial structure and functions. ATP synthase is a crucial hub in mitochondria, since it produces most of the energy needed to sustain cellular activity. It participates in shaping the structure of mitochondrial cristae and the permeabilization of the inner mitochondrial membrane, and it controls intracellular signalling pathways [13, 14]. Therefore, it could be hypothesized that on mammalian cells TB could induce mitochondrial dysfunction and consequent oxidative damage.

An alternative hypothesis was proposed by Zhu X *et al.*, [15] who observed and suggested that TB could act with mechanisms similar to bisphosphonates (BPs), i.e. by modulating RANKL and MAP kinase pathways. In summary, the goal of the present work was to better evaluate, also by using an in vivo model, the activity of TB on MPM cells.

## Materials and methods

### Mesothelioma cell lines

In this work, we employed commercially available immortalized cell lines (the malignant Mero-14, Mero-25, Ren, NCI-H28, MSTO-211H, and the non-malignant MeT-5A). MeT-5A was purchased from ATCC, Mero-14 and Mero-25 were kindly donated by Istituto Tumori of Genova (Italy); Ren was kindly provided Dr. Laura Moro, Universita` del Piemonte Orientale ‘A. Avogadro’, Novara, (Italy); NCI-H28 cells were kindly donated by collaborators of the Pharmaceutical Department of the University of Pisa (Italy); MSTO-211H were kindly donated by collaborators of the Barts Cancer Institute (London, UK). Culturing conditions are described in our previous paper [16]. All cell lines were routinely passaged every 1–2 weeks. Cells were grown at 37 °C and 5% CO_2_.

### Protein extraction and western blot

Protein extraction and western blots procedures are described in our previous paper [16]. The following primary and secondary antibodies, purchased from Proteintech Europe (Manchester, UK), were employed: BAX (50599-2-Ig BAX Rabbit Polyclonal antibody), β-tubulin (10068-1-AP Beta Tubulin Polyclonal antibody), Peroxidase-conjugated Affinipure Goat Anti-Mouse IgG(H + L) (SA00001-1) and Peroxidase-conjugated Affinipure Goat Anti-Rabbit IgG(H + L) (SA00001-2). We bought from Cell Signaling Technology (Danvers, MA, USA) the primary antibodies for ERK1/2 (#4695T, p44/42 MAPK (Erk1/2) (137F5) Monoclonal Rabbit), phospho-ERK1/2 (#4370S, Phospho-p44/42 MAPK (Erk1/2 - Thr202/Tyr204 (D13.14.4E) XP® Monoclonal Rabbit), p38 (#8690, p38 MAPK (D13E1) XP® Rabbit mAb), phospho-p38 (#4511, Phospho-p38 MAPK - Thr180/Tyr182 (D3F9) XP® Rabbit mAb). The primary antibody for farnesyl pyrophosphate synthase (FPPS) was by Bethyl Laboratories® (# A305-045 A Rabbit anti-FDPS Antibody).

### Restoring of cells viability

After seeding in 96-well plates, cells were incubated for 24 h at 37 °C and 5% CO_2_ and then treated with either vehicle, 1 µM of TB, 12.5 µM geranylgeraniol (GGOH), farnesol (FOH) (for NCI-H28 and MSTO-211H, 3.9 nM, for Mero-14 and Mero-25, 1.9 nM, and for MeT-5A and IST-Mes2 1 nM) alone or in combination. GGOH and FOH were purchased from Cayman Chemical Company (Ann Arbor, MI, USA). Proliferation was assessed 72 h after treatment by adding 3-(4, 5-dimethylthiazolyl-2)-2, 5-diphenyltetrazolium bromide (MTT) solution, and following the procedure described in our previous paper [16]. Three independent experiments were performed, each in duplicate. TB was purchased from Sigma Aldrich (catalog number: TA9491597867).

### Mitochondrial reactive oxygen species (mROS) measurements

Cells were seeded in 6-well plates and treated with TB at different time-points as reported in the figure legends. Next, cells were incubated for 30 min with the red fluorescent probe MitoSOX to detect the total ROS release from mitochondria. Fluorescence signal was measured on a confocal laser scanning microscopy Olympus FV3000 with a fixed exposure time among the different conditions.

### Mitochondrial membrane potential (ΔΨ_m_) measurements

Cells seeded on 24-mm glass coverslips, previously treated as indicated in the figure legends, were loaded with 20 nM tetramethylrhodamine, methyl ester (TMRM) for 30 min at 37 °C. Images were taken on an inverted confocal fluorescence microscope Olympus FV3000 equipped with a 60x oil objective. Images were taken every 5 s with a fixed exposure time. After 5 acquisitions carbonyl cyanide p-trifluoromethoxyphenylhydrazone (FCCP, 10 mM), an oxidative phosphorylation uncoupler, was added to collapse the electrical gradient established by the mitochondrial respiratory chain.

### Oxygen consumption rate (OCR) measurements

Cells were seeded on a 96-well SeaHorse microplate and treated as reported in the figure legends. Before the experiments with Mito Stress test kit (Agilent, CA, USA), the culture medium was replaced with 175 µl of Seahorse XF Base Medium pre-warmed at 37 °C, completed with 10 mM glucose, 1 mM Pyruvate and 2 mM glutamine, and the plate was incubated in a CO_2_ free incubator at 37 °C for 1 h. The following compounds were prepared: 2 µM oligomycin, 1 µM FCCP 1 µM, 1 µM Rotenone/Antimycin A (Rot/AA). A volume of 25 µL of compound was added to each injection port, and measurements were taken after each addition. OCR values were finally normalized to the number of cells per well by using the crystal violet method. Briefly, after measurements, cells were fixed with 2% paraformaldehyde for 15 min, stained with 0.1% crystal violet for 20 min and washed. Absorbance was measured at 595 nm. The different parameters were calculated as follow. The basal OCR was detected by the equation: OCR before addition of oligomycin – OCR after addition of Rot/AA. The ATP production as OCR before addition of oligomycin – OCR after addition of FCCP. The Maximal OCR was computed as the highest OCR after addition of FCCP subtracted from the OCR after addition of the mixture of Rot/AA.

### Ca^2+^ measurements

Cells were seeded in 13-mm glass coverslips and then transfected with cytosolic (cytAEQ) and mitochondrially targeted (mtAEQ) aequorin. After 24 h, cells were treated as reported in the text and then incubated for 2 h in KRB (Krebs-Ringer modified buffer,135 mM NaCl, 5 mM KCl, 0.4 mM KH2PO4, 1 mM MgSO4, 5.5 mM glucose, and 20 mM Hepes, pH 7.4) at 37 °C supplemented with coelenterazine and transferred to the perfusion chamber of a luminometer. Agonist was added to the same medium, as specified in the figure legends. The experiments terminated by lysing cells with 100µM digitonin in a hypotonic Ca^2+^-rich solution (10mM CaCl_2_), to discharge the remaining aequorin pool. Finally, the light signal was collected and calibrated into [Ca^2+^] values, as previously described [17].

### Cells and retroviral infection

The Human Pleural mesothelioma epithelioid Ren cell line, isolated and characterized by Dr Albelda SM (University of Pennsylvania, Philadelphia, PA, USA) [18], were transduced with L-LUC-IN2, a bicistronic retroviral vector coding for both the firefly luciferase and the NeoR genes, to allow the in vivo monitoring of tumour burden (see paragraph *Mice and vivo experiments*).

### Mice and in vivo experiments

Six-week-old female Non-Obese Diabetic Severe Combined Immunodeficient (NOD-SCID) mice were purchased from ENVIGO (San Pietro al Natisone, Udine, Italy). The animals were housed in pathogen-free environment, and all experiments were performed in accordance to the National Regulation on Animal Research Resources and approved by the Review Board of the IRCCS Policlinico San Martino, Genoa, Italy, and Italian Ministry of Health (n° 462/202PR, Dl.vo 4/04/2014 n 26). Twenty NOD-SCID female mice were pseudo-orthotopically inoculated by intra-peritoneal (i.p.) injections of 5 × 10^6^ luciferase transduced Ren cells in 0.5 mL of DMEM medium. Mice were monitored for disease symptoms every other day (starting from one weeks after tumour challenge) by visual inspection and sacrificed by CO_2_ asphyxiation at Human End Points to prevent or alleviate pain and/or distress. Ten days after cell inoculation tumour take-rate in the peritoneal cavity was evaluated by two Bioluminescent imaging (BLI) acquired two days apart. After anesthetization by inhalation of isoflurane (Induction phase: 3,5% in O_2_, Maintenance phase: 1–2% in O_2_) continuously dispensed for the duration of the procedure, mice were i.p. injected with 0.3 mL of 15 mg/mL D-luciferin. Tumour dimension and localization of luminescent cells were monitored for each animal using the In Vivo Imaging System (IVIS®) Lumina III series (PerkinElmer Inc, MA, USA). Region of interest (ROI) was identified and quantified as total photon counts using Living Image software (PerkinElmer Inc, MA, USA) and expressed as photons/seconds/cm^2^/sr. Mice were then weighed and divided into two groups of ten animals each, having similar ROI mean and distribution, and randomly assigned to treatment and control groups. TB was dissolved in DMSO at 26 mg/mL and diluted to 0,8 mg/ml in saline 1% glycerol formal immediately before i.p. injection. TB was administered twice a week for four weeks, by i.p. injection at 4 mg/kg in saline 1% glycerol formal. Untreated animals were i.p. dosed with the same volume of saline 1% glycerol formal. Tumour growth was monitored by two further IVIS acquisition.

### Statistical analysis

The statistical analyses were carried out with GraphPad Prism 8.0 software (Microsoft Inc, Redmond, WA, USA) and with Statgraphics Centurion XVI (version 16.1.11; StatPoint Technologies Inc., Warrenton, VA, USA), and 0.05 was employed as the statistical threshold of significance. Regarding the in vivo experiments, overall survival (OS) curves were estimated for each group using the Kaplan-Meier method, the Log-rank (Mantel-Cox) test was used to statistically compare the curves.

## Results

### In vitro evaluation of possible biological effects of TB

#### Evaluation of the role of TB in affecting proliferation and apoptotic pathways

Because our previous results suggested an antiproliferative effect of TB on MPM cells, here we studied the phosphorylation of ERK 1/2, a known ubiquitous player whose activation has a key-role in inducing cell-proliferation. Moreover, we also evaluated the phosphorylation of p38, whose activation induces stress-counteracting effects such as cell differentiation, growth arrest, and apoptosis [19]. Indeed, the constitutive activation of p38 is a common event in MPM cells [20, 21].

**Fig. 1:**
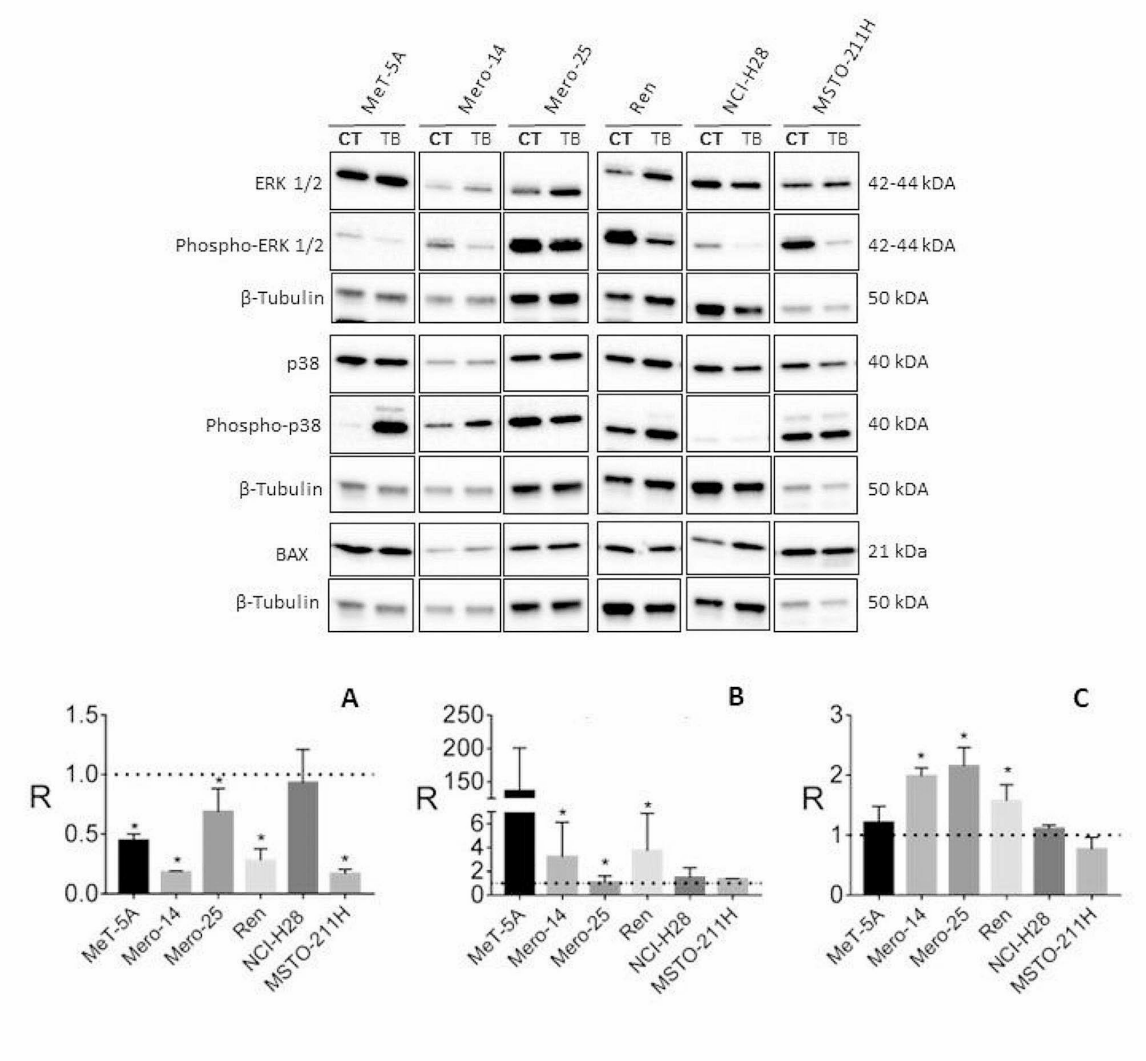
Effects of TB on ERK1/2 and p38 phosphorylation and BAX. Western blot analysis of the protein expression induced by TB (1 μM) at 24 h (for ERK 1/2 and p38) or 48 h (for BAX) post- treatment. β-Tubulin was used as reference. The picture is representative of one of two experiments, while the histograms were generated by quantifying blots from both experiments. The histograms report (Y-axis) the ratio (R) of the measurements obtained with Image Lab Software for **(A)** ERK 1/2 (Phospho-ERK/total ERK), **(B)** p38 (P-p38/total p38), and **(C)** BAX (BAX/β-Tubulin) relative to the controls (DMSO, the vehicle, shown as a dotted line) in MeT-5A, Mero-14, Mero-25, Ren, NCI-H28, and MSTO-211H cell lines (X-axis). Data are expressed as mean ± standard error and the statistical significance (*p*<0.05) of the comparisons between controls and treatments is indicated by the asterisk (*)

Interestingly, with the only exception of NCI-H28 cell line, we observed that, after 24 h of treatment, TB significantly suppressed the phosphorylation of ERK 1/2. Moreover, in Met-5A, Mero-14, and Ren cell lines TB caused an enhancement of the phosphorylation of p38. Finally, because our previous results suggested that TB could induce the activation of caspases [3], here we studied the expression of the pro-apoptotic protein BAX and, 48 h post-treatment, we found a slightly increased expression in Mero-14, Mero-25, and Ren cell lines (Fig. 1).

#### Evaluation of the role of TB in affecting the mitochondrial function

It has been hypothesized that TB could act at the mitochondrial level by affecting ATPase-ATP synthases and by activating the apoptotic cascade via BAX. Thus, we next moved to explore whether TB could somehow affect the mitochondrial functioning and dynamics. First, following the treatment with TB, we measured the mitochondrial and cytosolic Ca^2+^ transients (as markers of the mitochondrial function) after agonist stimulation capable of inducing the Ca^2+^ release from the endoplasmic reticulum (ER). Based on our previous work, showing that all these cellular functions may be regulated across different time ranges [22], these measurements were performed at different time points to detect the possible presence of different effects. The Ren cell line was employed as a model because it showed a very high activation of BAX and it was already manipulated in our laboratories, as luciferase transduced cell line, to be ready for future uses in the planned in vivo experiments.

**Fig. 2:**
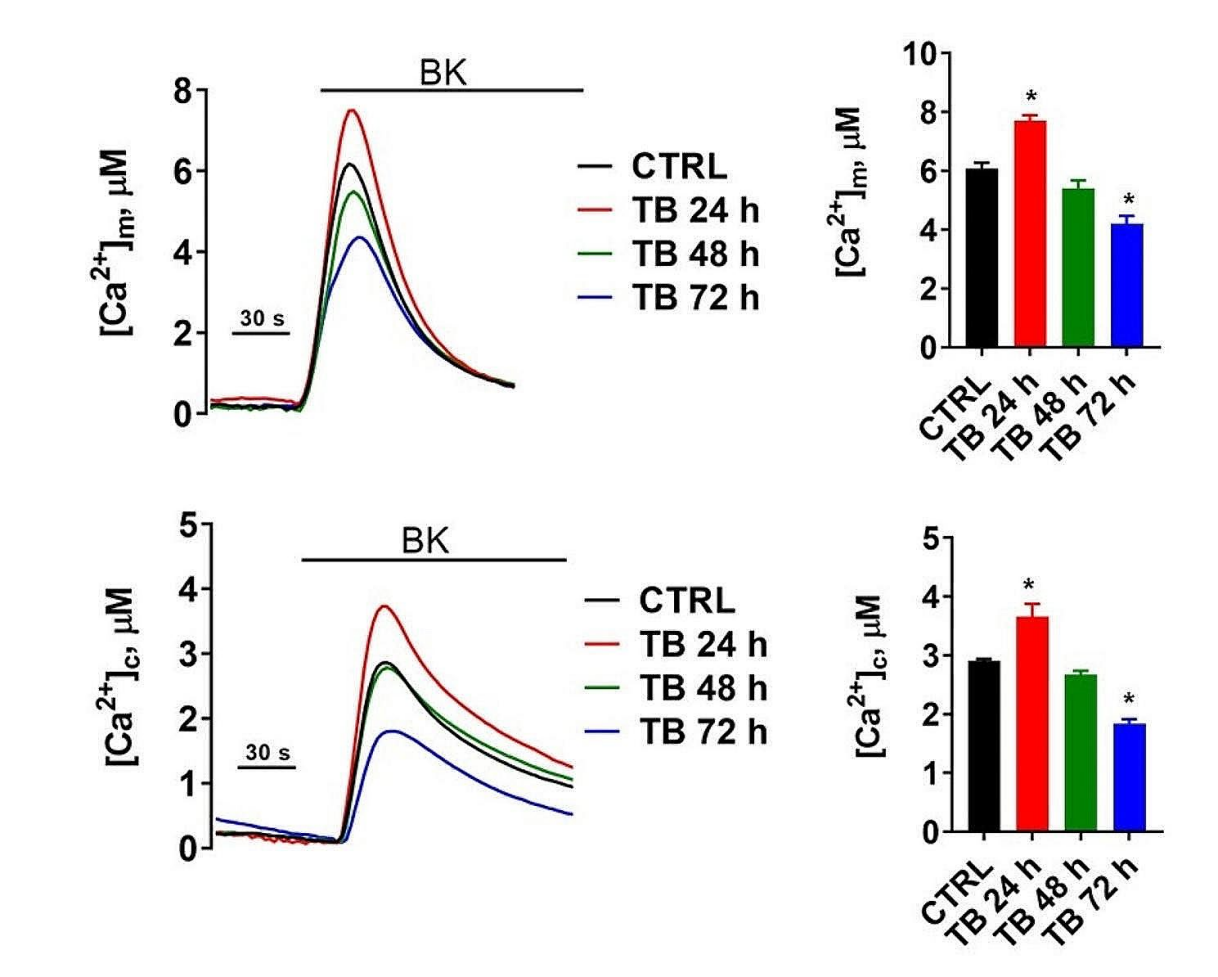
TB affects the Ca^2+^ transmission from ER to mitochondria. Ren cells were transduced with aequorin targeted to the mitochondrial (upper panel) and cytosolic (lower panel) compartments and treated with TB at different time points. To evoke the Ca^2+^ release from the ER and the consequent uptake in the mitochondria and cytosol, cells were stimulated with 1 µM Bradykinin (BK). Representative traces of Ca^2+^ dynamics are shown on the left

**Fig. 3:**
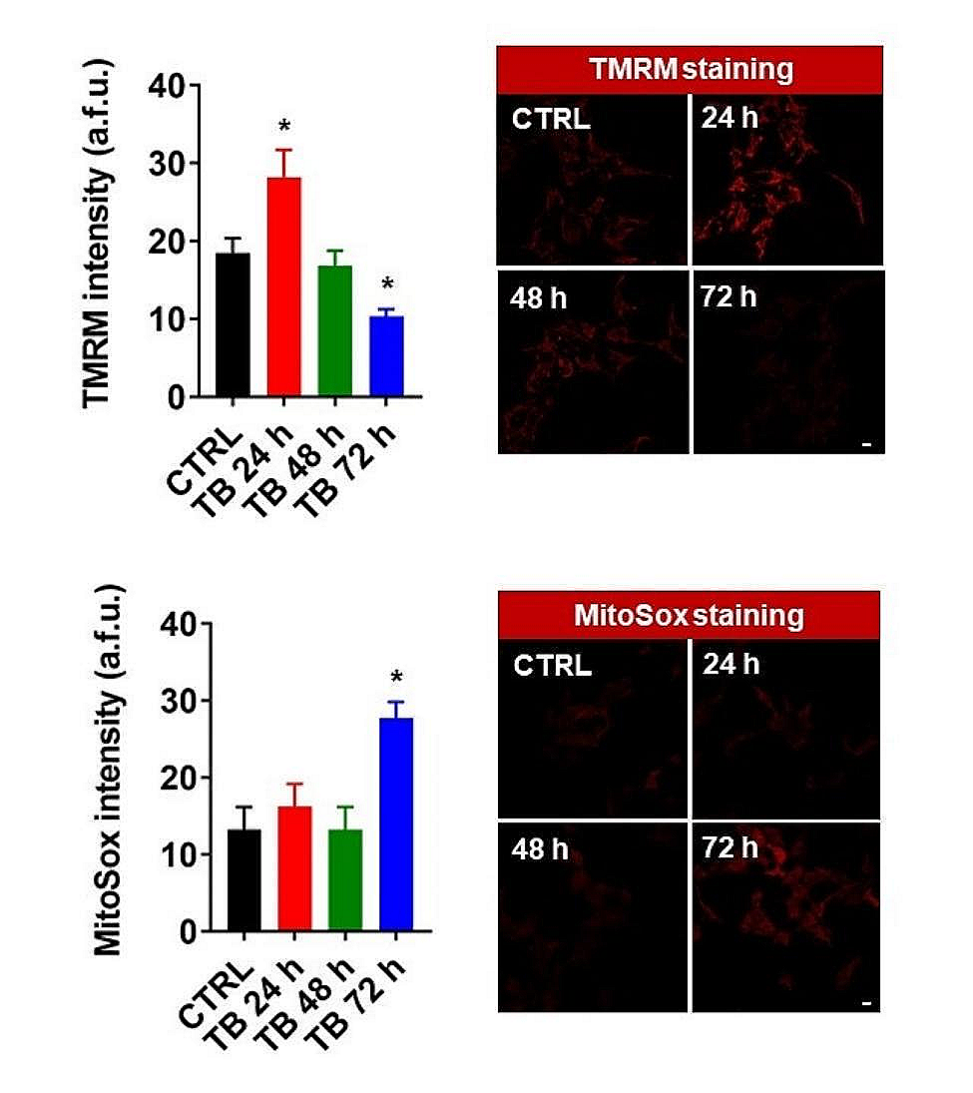
TB regulates the mitochondrial functioning. Ren cells were treated with TB at different time points and then loaded with the potentiometric dye TMRM to detect the levels of the mitochondrial membrane potential. The production of mitochondrial reactive oxygen species was analyzed by using the mitochondrial superoxide indicator MitoSox. Scale Bar: 10 μm

**Fig. 4:**
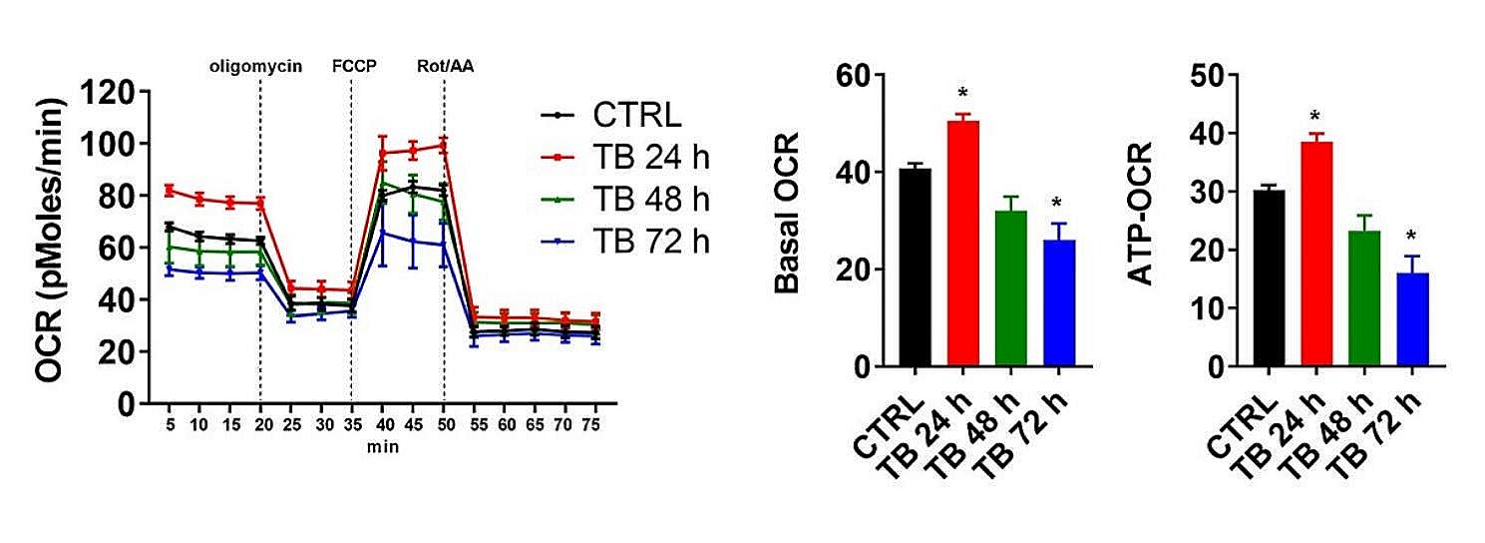
TB modulates the mitochondrial metabolism. OCR traces of control (black line) and TB-treated Ren cells expressed as picomoles of O_2_ per minute, under basal conditions and after the injection of oligomycin, FCCP, and Rot/AA. Quantifications of basal (Basal OCR) and ATP-related respiration rates (ATP-OCR) were calculated from OCR traces

As reported in Fig. 2, we found that treatment with TB at 24 h induced a simultaneous increase in both mitochondrial and cytosolic Ca^2+^ dynamics. At 48 h the levels detected were similar to those detected in untreated conditions and at 72 h we observed a significant reduction in Ca^2+^ homeostasis in both intracellular compartments. Moreover, we found that TB treatment for 72 h provoked the loss of ψ_m_ and a parallel increase of ROS production (Fig. 3). Having found that TB modulated the intracellular Ca^2+^ dynamics, we asked whether this compound could also modulate the mitochondrial metabolism. As reported in Fig. 4, 24 h of TB treatment increased the mitochondrial respiration and ATP production, while prolonged exposure to TB provoked a progressive reduction (48 h) and loss (72 h) of these mitochondrial parameters.

#### Evaluation of the role of TB in affecting the mevalonate pathway

**Fig. 5:**
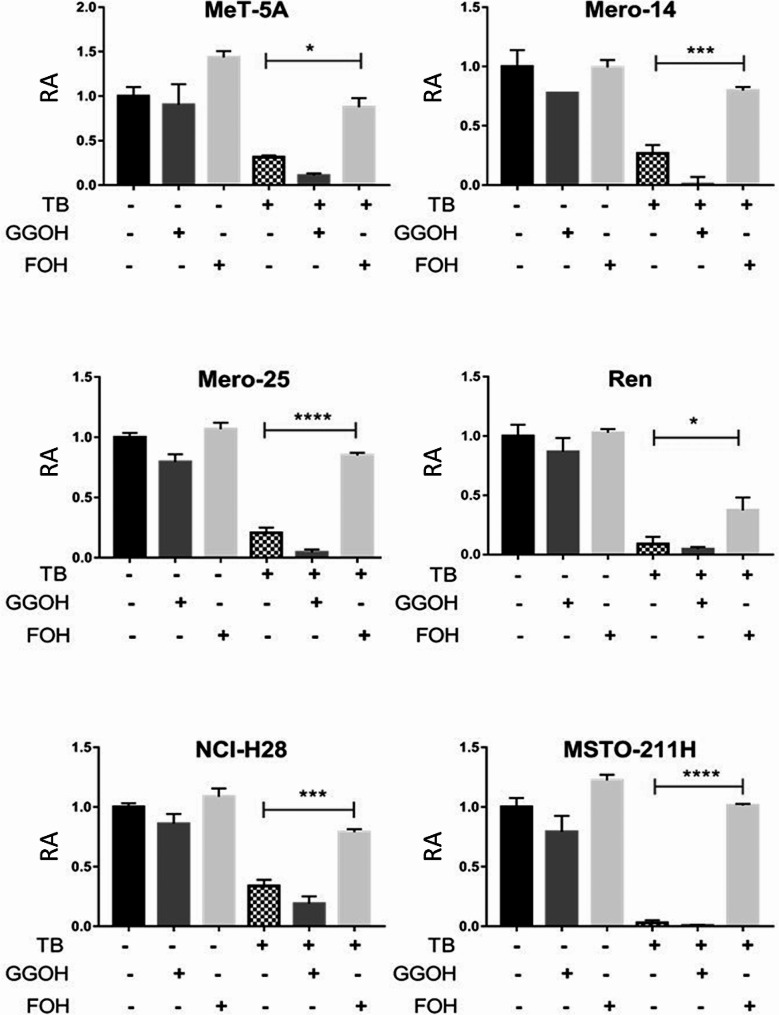
FOH and GGOH-mediate restoration of TB cytotoxicity in MPM cells. Cells were treated with the mixture of molecules indicated by the “+” signs reported in the lower parts of the plots. Y-axis shows, relative to the controls, the absorbance at 595 nm obtained with the MTT assay as a proxy of the cell viability, and expressed as relative absorbance “RA”. Measurements were carried out at 72 h after the treatment. Controls consisted of a treatment with vehicle only (DMSO). Error bars report the mean ± standard errors of three independent experiments. The statistical significance of the comparisons of each combination with the controls is indicated by asterisks (*), where *= *P* < 0.05; **= *P* < 0.01; ***= *P* < 0.001

We also hypothesized that the cytotoxicity exerted by TB in MPM cells could be ascribed to the same mechanisms of action known for BPs. Therefore, we next analysed whether TB could induce an inhibition of the mevalonate pathway, a well-known target of BPs [23]. Then, TB-treated cells were cultured in the presence or absence of the mevalonate compounds, GGOH and FOH. As shown in Fig. 5, the addition of FOH, but not GGOH, completely abolished the cytotoxic effects of TB, significantly restoring the viability of the tested cell lines. This effect could be ascribed to the inhibition of the activity of FPPS, a key enzyme in the mevalonate acid metabolic pathway and major molecular target of nitrogen-BPs [24]. The reduction of activity of FPPS was not due to an increased degradation, as we did not observe a reduced amount of the protein, measured at 0 and 24 h after the treatment with TB by western blots (Figure S1).

### In vivo evaluation of TB by using an MPM-xenografted mouse model

Based on the in vitro results, we tested if treatment with TB could affect MPM tumour growth in an in vivo mouse model. Twenty NOD-SCID mice pseudo-orthotopically inoculated with human MPM Ren cells were evaluated by BLI for tumour take rate and dimension, stratified into two groups of ten animals each and randomly assigned to treatment and control groups. TB was administered by i.p. injection at 4 mg/kg, twice a week for 4 weeks starting from day 11 after tumour transplant. Untreated animals were subcutaneously dosed with solvent. We chose i.p. administration to increase the TB local concentration around the tumour site, a method that eventually will be easily translated in clinical settings. Anti-neoplastic drug efficacy was evaluated by comparing OS time from tumour inoculation in TB treated group *vs*. control group. We did not use surrogate IVIS parameters, because the BLI values are reduced in case of peritoneal bleeding. This is a side effect frequently observed in advanced stages of peritoneal mesothelioma leading to an underestimation of the real tumour volume.

**Fig. 6:**
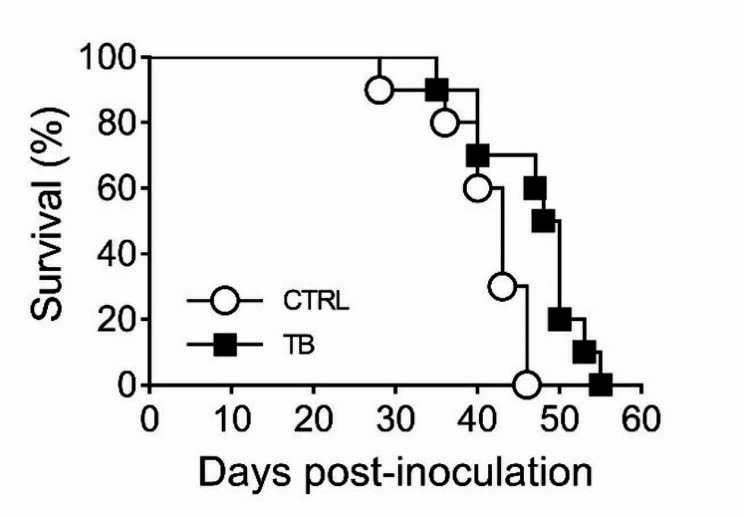
NOD-SCID female mice were injected i.p. with Ren cells. Mice received administrations of solvent or 4 mg/kg of TB twice a week. Differences in survival were statistically significant, as determined by log-rank analysis (*p* < 0.05)

Kaplan-Meier survival curves showed increased OS for TB treated group *vs.* vehicle control group (log-rank (Mantel-Cox) test, χ^2^_1_ = 7.117; *P* = 0.0076) [Fig. 6], although the tumour growth apparently did not change in either group as evaluated by BLI. This was likely due to the haemorrhagic ascites (affecting the BLI) observed more frequently in control group than in the TB group. In fact, by 7 weeks from MPM cell inoculation, 10/10 mice treated with vehicle and 5/10 mice treated with TB were dead.

## Discussion and conclusion

TB is typically employed as surfactant, preservative, and antimicrobial agent for topical use only. It was reported also as an antifungal [5] and anthelmintic [4] agent. Moreover, similarly to BPs, TB was found to have an antiresorptive effect on bone [15], by blocking the RANKL-induced activation of the MAPK signalling pathways (ERK 1/2 and p38). In a previous work of drug screening [3], TB showed to be the most effective in activating caspases 3/7, and in inhibiting the proliferation and the clonogenicity of an extended panel of MPM cell lines. Moreover, it also affected the viability and growth of 3D spheroids of patient derived MPM cells [3]. Most of the mechanisms related to the biological activities of TB are only partially known. Therefore, we departed from previous evidence and generated some hypotheses to be verified in the context of MPM.

Following the observed activity on the MAPK signalling pathway, it could be hypothesized that part of the anticancer activity of TB on MPM has to be ascribed to the regulation of ERK1/2 and p38 pathways. In fact, it is well known that these molecules are frequently activated in MPM [20, 21, 25]. ERK1/2 has a key-role in inducing cell proliferation while p38 becomes activated when cells need to counteract stress stimuli. This response is usually elicited by inducing cell differentiation, apoptosis, and growth arrest. In agreement with this idea, we observed that TB significantly suppressed the phosphorylation of ERK 1/2 and enhanced the phosphorylation of p38. The effect of TB on apoptosis could be also due, at least in part, to the induction of the pro-apoptotic protein BAX. These findings corroborate our previous results and agree with the activation of MAPK signalling pathways observed in the study by Zhu *et al.* [15] above mentioned. During apoptosis, BAX (together with the pro-apoptotic protein BAK) is activated and accumulates on the outer mitochondrial membrane to cause its permeabilization, with a consequent release of apoptotic factors in the cytosol, thereby activating the apoptotic cascade [26]. It has been demonstrated that to provoke its pro-apoptotic effects, BAX also modulates the Ca^2+^ homeostasis, by controlling the Ca^2+^ transmission between the ER and the mitochondria and by regulating the mitochondrial ψ_m_ and the generation of ROS [27]. Our results suggest that TB could also modulate the ER-mitochondrial Ca^2+^ transmission, as demonstrated by increased accumulation of Ca^2+^ in both mitochondria and cytosol. Furthermore, the fact that the Ca^2+^ variations observed are progressively lost over time (48 h and 72 h) could indicate that the mitochondrial apoptotic pathway is executed. This possibility was confirmed when we measured the levels of the ψ_m_ and the ROS production. Indeed, we found that TB treatment for 72 h provoked the loss of ψ_m_ and a parallel increase of ROS production, two aspects highly related to the apoptotic machinery [28] and in agreement with the observed activation of caspases 3/7.

Notably, apoptosis is a cellular event which requires high amount of ATP. It has been demonstrated that the Ca^2+^-transmission from ER to the mitochondria is a key event to activate the mitochondrial bioenergetic and the ATP production [29, 30]. Having found that TB modulated the intracellular Ca^2+^ dynamics, we asked whether this compound could also modulate the mitochondrial metabolism. The mitochondrial respiration and ATP production were increased after 24 h of TB treatment, while prolonged exposure of TB provoked a progressive reduction (48 h) and loss (72 h) of these mitochondrial parameters, being the results overall in agreement with what observed when ψ_m_ and ROS production were measured.

The last in vitro analysis was aimed to verify whether the cytotoxicity exerted by TB in MPM cells could be also ascribed to the involvement of the mevalonate pathway. FOH is the common substrate for squalene and GGOH [31] and both these molecules should be rescued once FOH is administered for bypassing the FPPS inhibition. However, we did not observe a reduced cytotoxicity of TB when co-administered with GGOH, rather, we found an enhanced cytotoxicity. Therefore, we could speculate that the cytotoxicity of TB could be mainly due to the reduced formation of squalene and that the observed potentiation of cytotoxicity by the coadministration of TB with GGOH could be ascribed to a regulatory feedback mechanism. As a matter of fact, it has been shown that GGOH is an inhibitor of the 3-hydroxy-3-methylglutaryl-CoA reductase for the upstream production of mevalonate acid [32]. Further research on the action of TB on the mevalonate pathways is fully justified.

Overall, these data were intriguing and encouraged further experimentation on an in vivo system. Interestingly, mice treated intraperitoneally with TB showed a statistically significant prolonged survival compared to controls. Thus, we could hypothesize that TB treatment affected the tumour biology eliciting a better response of the xenografted mice. However, the toxicity associated with the in vivo i.p. administration of TB will need careful studies. Rather, the strength of TB for MPM could consist in its being a mitochondrial uncoupler, a putative pivotal role suggested by our results that lays the groundwork for further investigations on the use of uncouplers in MPM. Indeed, some of the effects we observed in MPM cells after administration of TB resemble a phenotype commonly associated with the anti-cancer effects of mitochondrial uncouplers [33]. A large body of evidence supports the therapeutic use of mitochondrial uncouplers to treat metabolic diseases (metabolic associated fatty liver disease, non-alcoholic hepato-steatosis, insulin resistance, type 2 diabetes) and cancer, and to date, some of them are in clinical trials for cancer, both as single agents and in combination therapies [33, 34]. However, an evaluation of mitochondrial uncouplers as anti-cancer agents in a model of MPM, both in vitro and in vivo, is still lacking. Further studies about this likely role of TB could address these gaps in knowledge and help progression of such small molecules into cancer clinical trials.

We are aware that TB was approved in humans for topical uses. For a systemic administration, a brand-new experimentation should be carried out departing from phase-1, denying our starting goal of detecting drugs ready-from-the-shelf, to be promptly used for MPM patients. However, TB showed to be one of the most active drugs among the 1170 tested in the library and a practical use could be still envisioned in the context of the hyperthermic intrathoracic chemotherapy. In fact, a cisplatin-based chemotherapy (alone or in combination) administered at 42 °C for 60 min after cytoreductive surgery (as part of a multimodal treatment) proved to be safe and effective in improving disease-free interval and overall survival of MPM patients [35]. The addition of TB to this type of treatment could be warranted in specific patients. In conclusion, the present work provided some hints on various biological mechanisms underlying the activity of TB and provided evidence that TB is a candidate for novel strategies in the fight against MPM.

### Electronic supplementary material

Below is the link to the electronic supplementary material.

**Supplementary Material 1**

**Supplementary Material 2: FPPS expression in non-malignant MeT-5A and MPM cells.** Western blot analysis of the expression of FPPS, a key enzyme of mevalonate pathway, after 24 hours of incubation with TB 1 μM. GAPDH was used as reference. The picture is representative of one of two experiments performed, while the histograms were generated by quantifying blots from both the experiments. The histograms report (Y-axis) the ratio (R) of the measurements obtained with Image Lab Software for FPPS relative to the controls in MeT-5A, Mero-14, Mero-25, Ren, NCI-H28 and MSTO-211H cell lines (X-axis). Controls consisted in a treatment with the vehicle only (DMSO) and are reported as dotted line. Data are expressed as mean ± standard error and the statistical significance of the comparisons between controls and treatments is indicated by asterisk (*), where =P<0.05; *=P<0.01; ***=P<0.001, compared to control treatment set to 1 (dotted line).

## Abbreviations

BLI: Bioluminescent imaging
BK: Bradykinin
BP: Bisphosphonates
ER: Endoplasmic reticulum
FCCP: Carbonyl cyanide p-trifluoromethoxyphenylhydrazone
i.p.: Intro-peritoneal
IVIS®: In vivo imaging system
mROS: Mitochondrial reactive oxygen species
MPM: Malignant pleural mesothelioma
NOD-SCID: Non-obese diabetic severe combined immunodeficient
OCR: Oxygen consumption rate
OS: Overall survival
ROI: Region of interest
ROS: Reactive oxygen species
TB: Thonzonium bromide
TMRM: Tetramethylrhodamine, methyl ester
ψ_m_: Mitochondrial membrane potential
