# Supplementary material for: Thonzonium bromide inhibits progression of malignant pleural mesothelioma through regulation of ERK1/2 and p38 pathways and mitochondrial uncoupling
Source: Cancer Cell Int. 2024 Jun 29;24:226. doi: 10.1186/s12935-024-03400-7 (PMC11218145; doi:10.1186/s12935-024-03400-7)
Supplement: Supplementary file 1 — Supplementary Material 1 [file 12935_2024_3400_MOESM1_ESM.pdf]

ORIGINAL RAW WESTERN BLOT DATA

FIGURE 1: ERK1/2 - Thonzonium Bromide (TB)

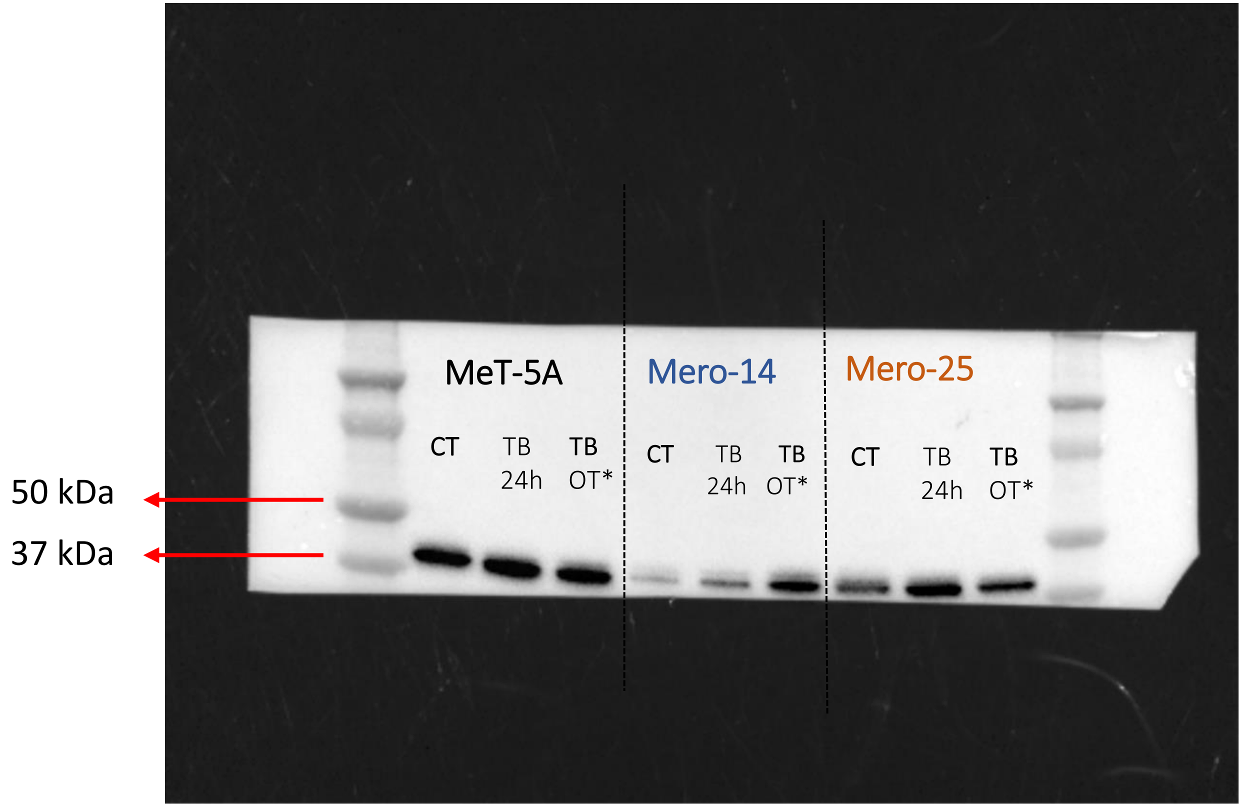

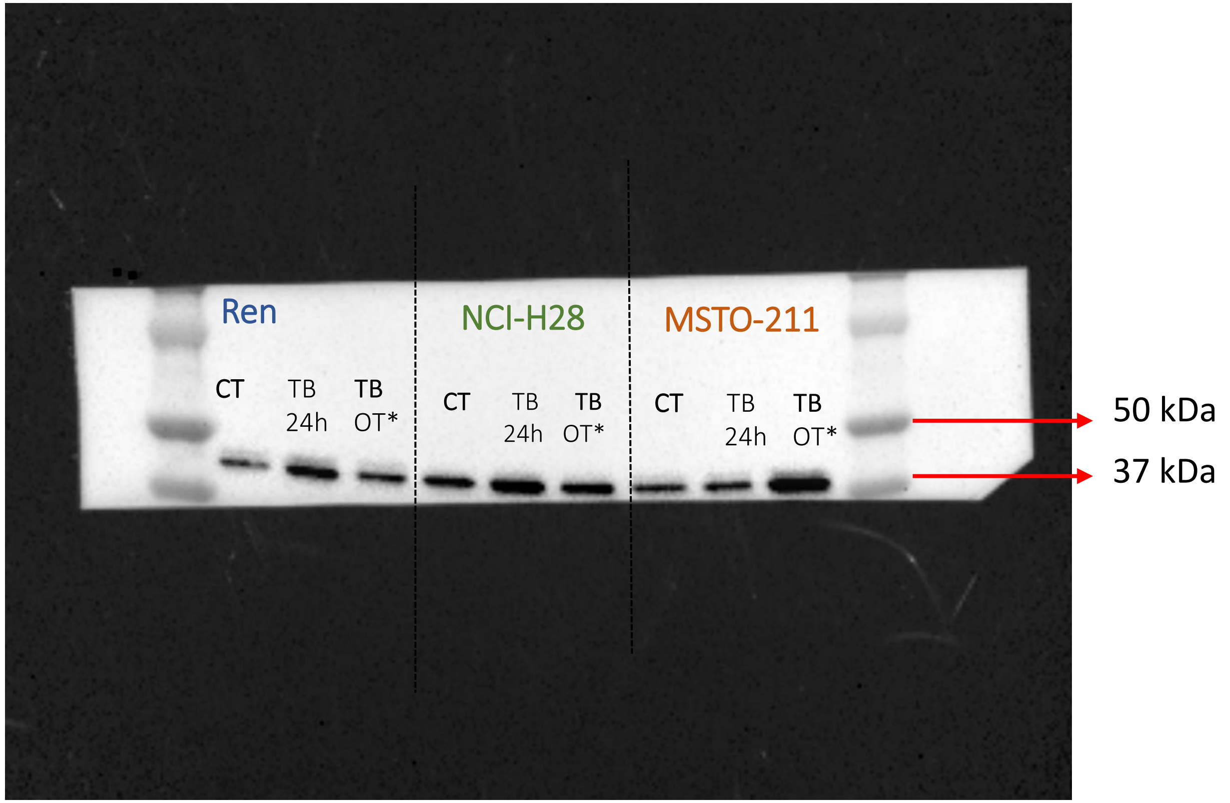

OT*: Other time point, not of interest

FIGURE 1: phospho-ERK1/2 -Thonzonium Bromide (TB) 24h

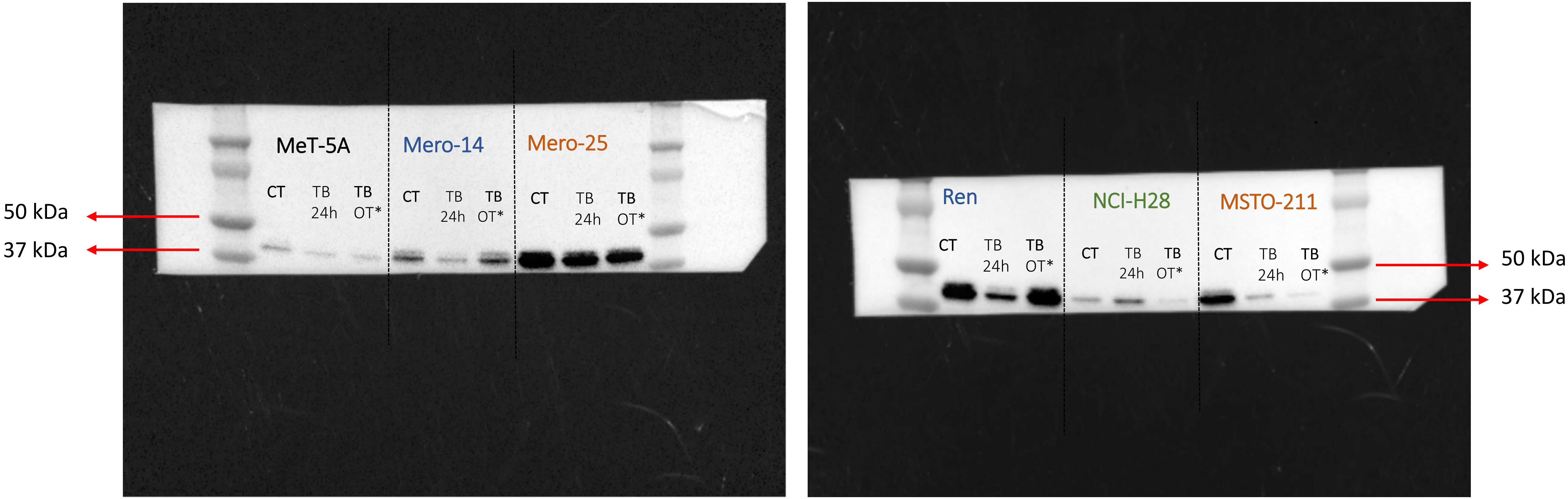

OT*: Other time point, not of interest

<u>FIGURE 1</u>: ß-Tubulin (ERK1/2 and phospho ERK1/2) - Thonzonium Bromide (TB) 24h

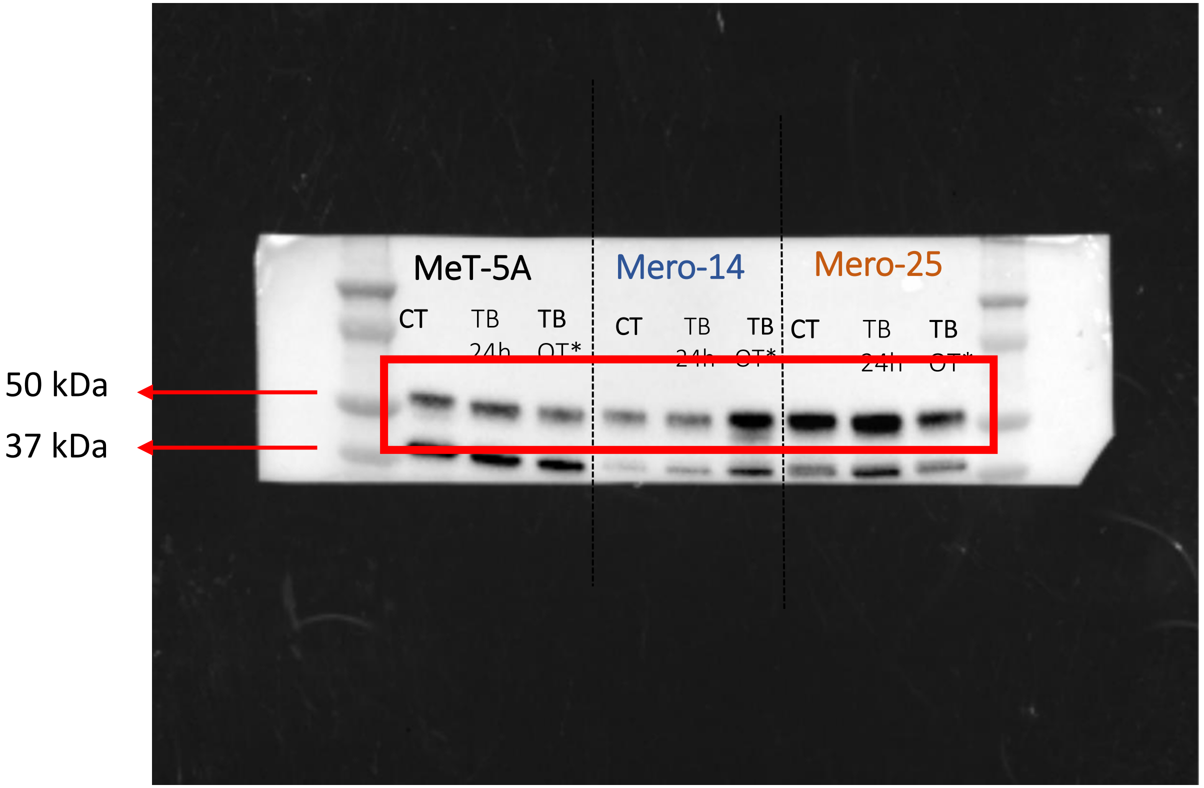

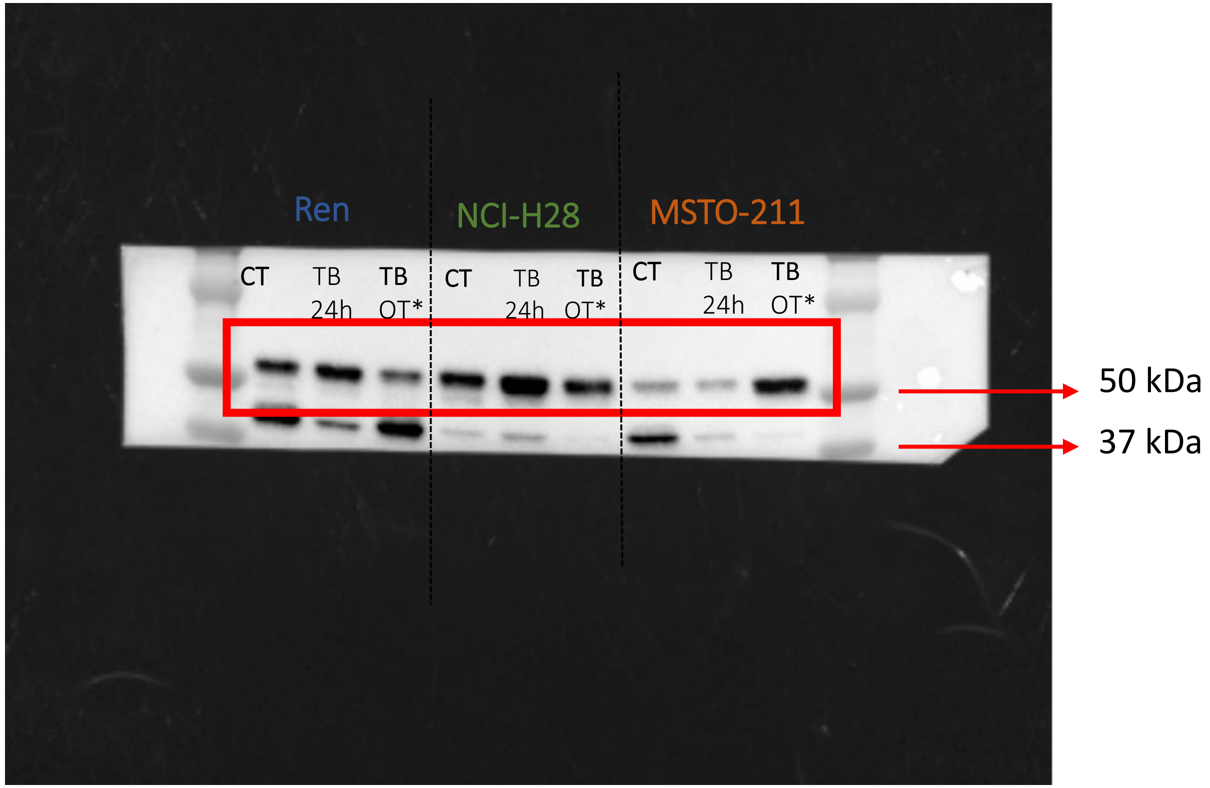

OT*: Other time point, not of interest

Figure 1: P38 - Thonzonium Bromide (TB) 24h

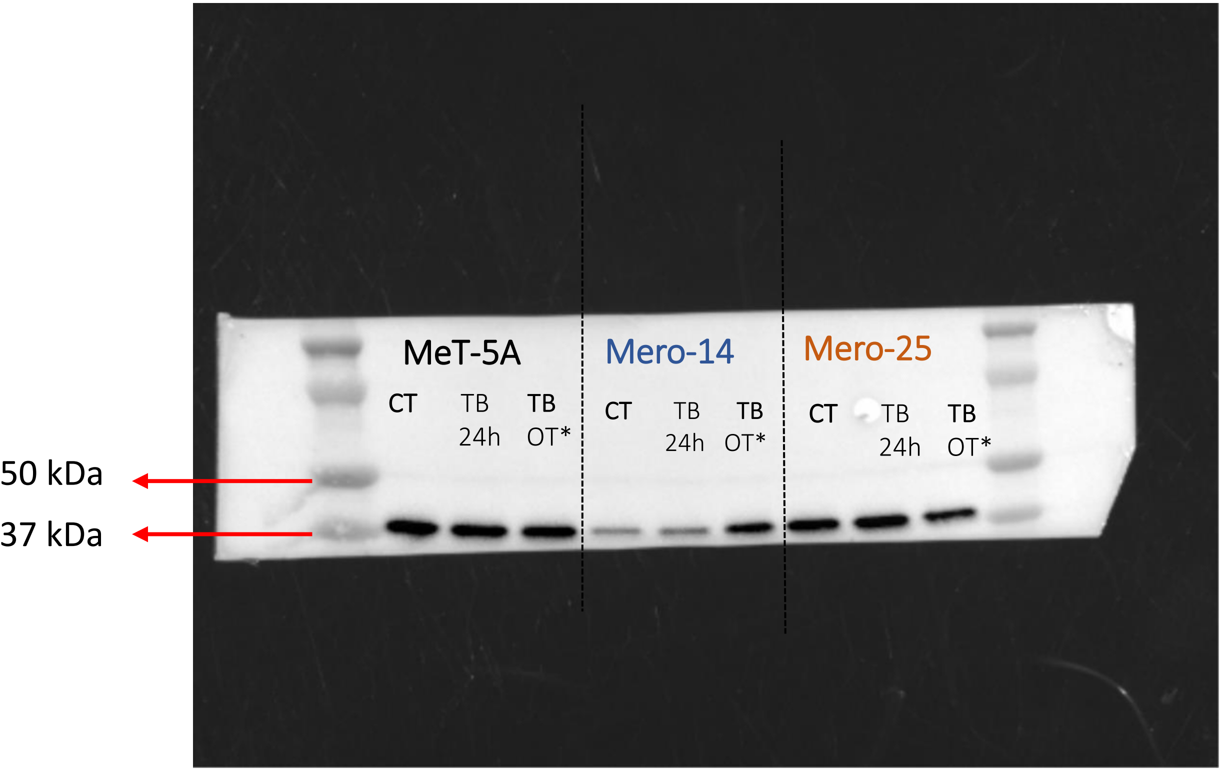

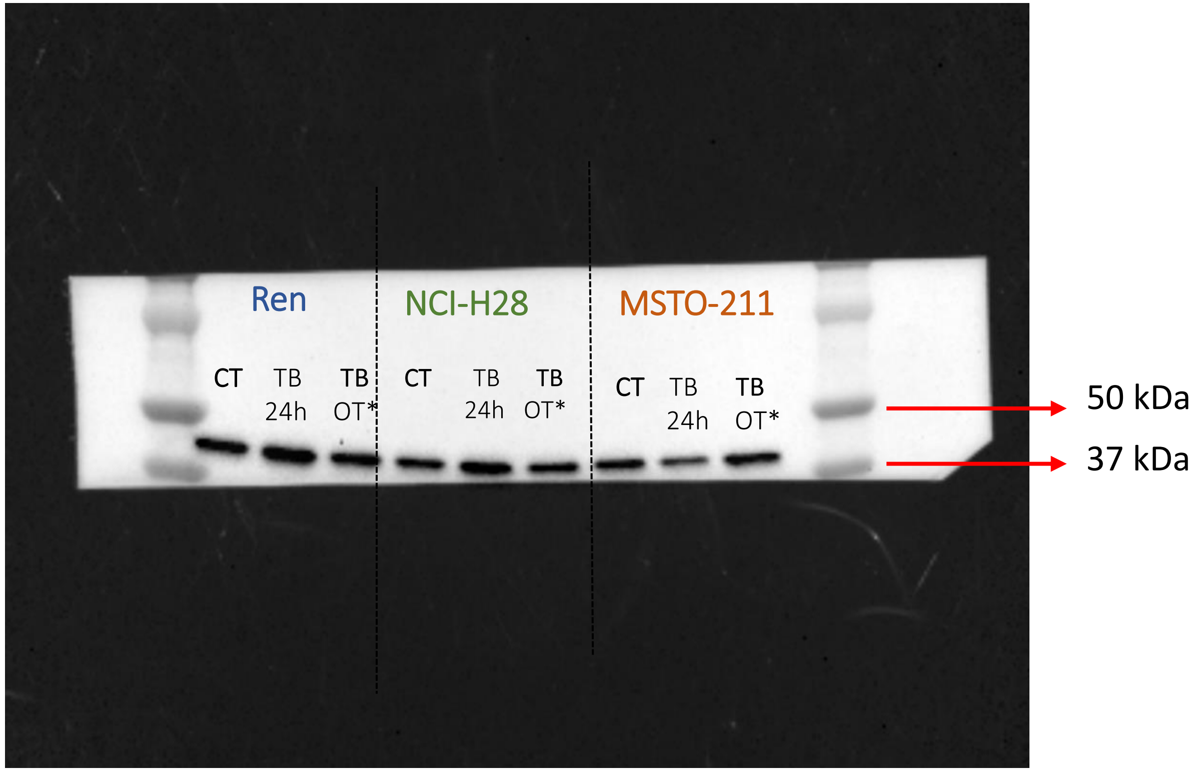

OT*: Other time point, not of interest

Figure 1: phospho-P38 - Thonzonium Bromide (TB) 24h

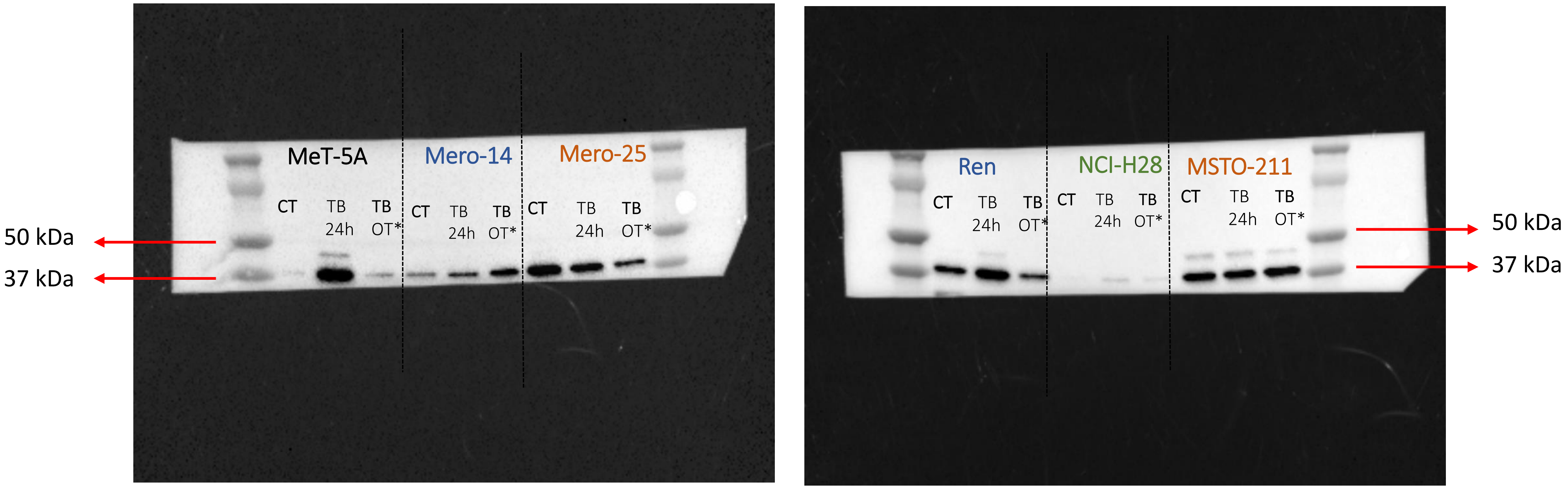

OT*: Other time point, not of interest

Figure 1: ß-Tubulin (P38 and phospho-p38) - Thonzonium Bromide (TB) 24h

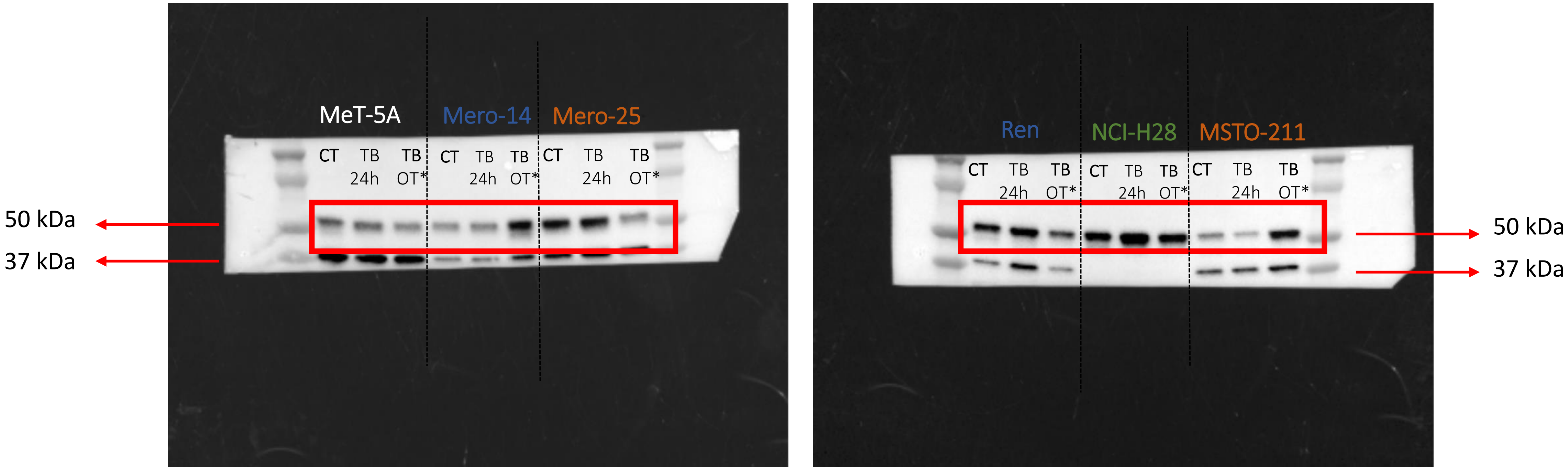

OT*: Other time point, not of interest

Figure 1: BAX- Thonzonium Bromide (TB) 24h

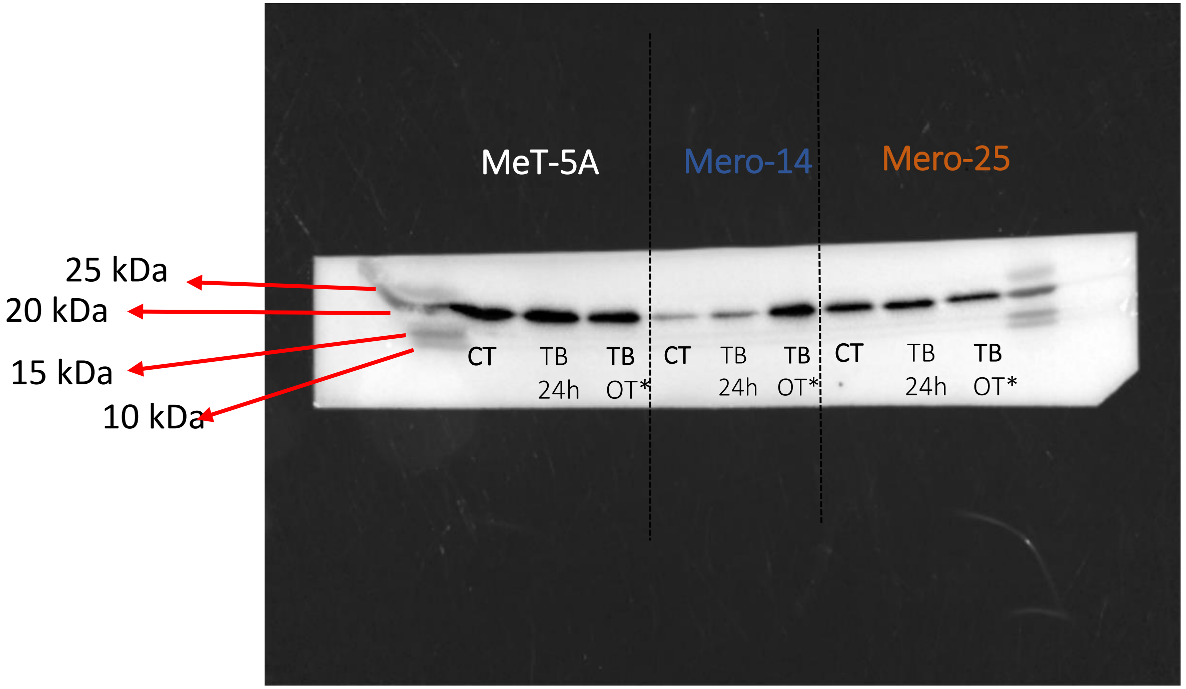

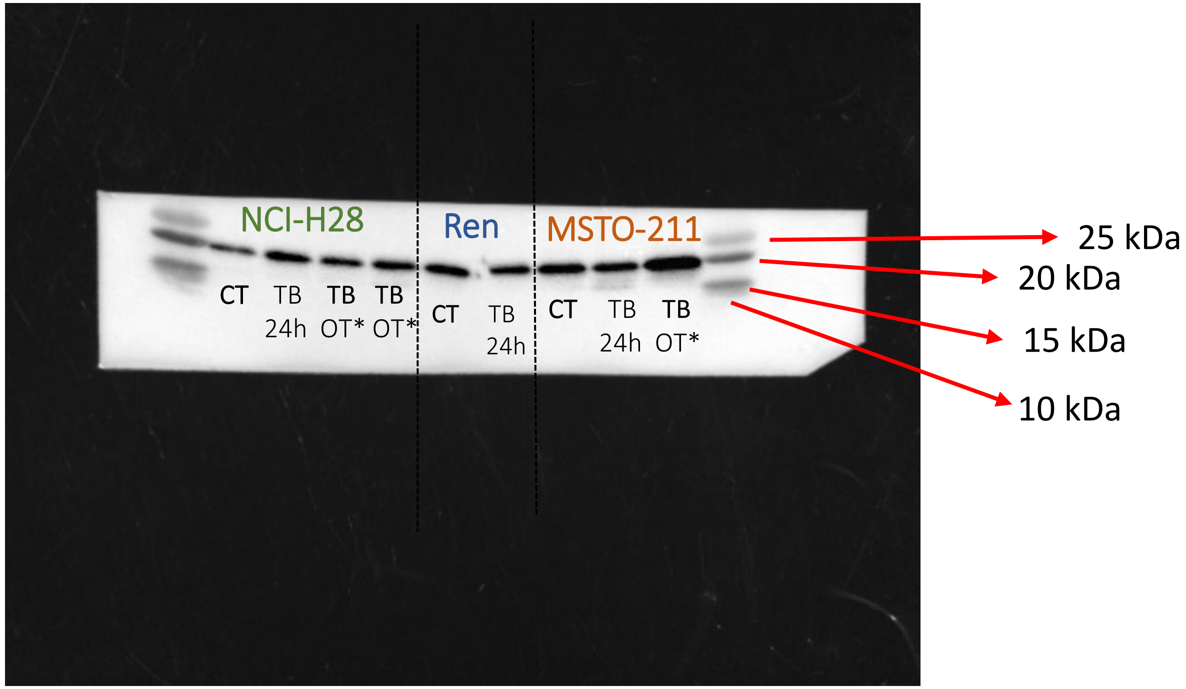

OT*: Other time point, not of interest

Figure 1: ß-Tubulin (BAX) - Thonzonium Bromide (TB) 24h

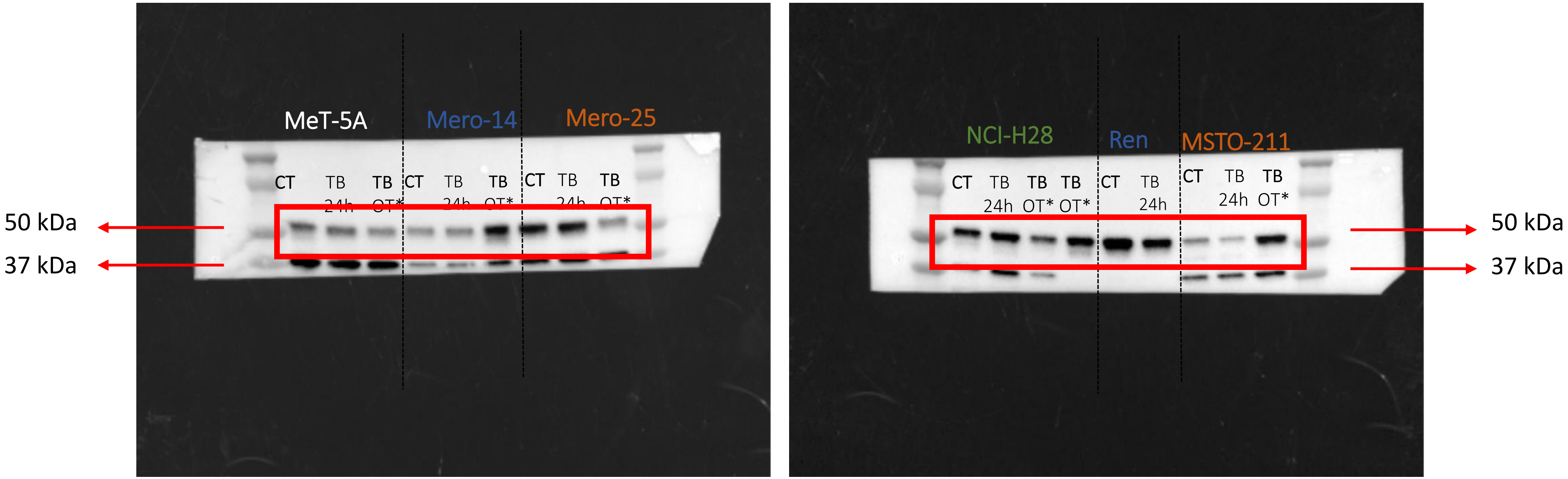

OT*: Other time point, not of interest

Figure S1: FPPS - Thonzonium Bromide (TB) 24h

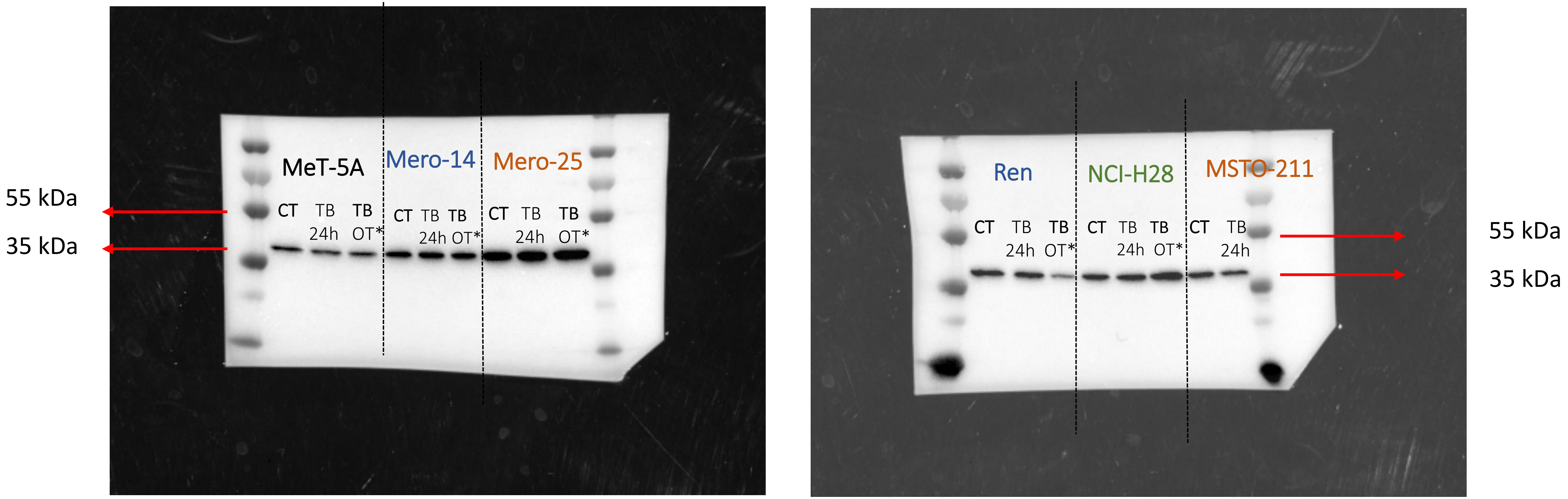

OT*: Other time point, not of interest

Figure S1: GAPDH (FPPS) - Thonzonium Bromide (TB) 24h

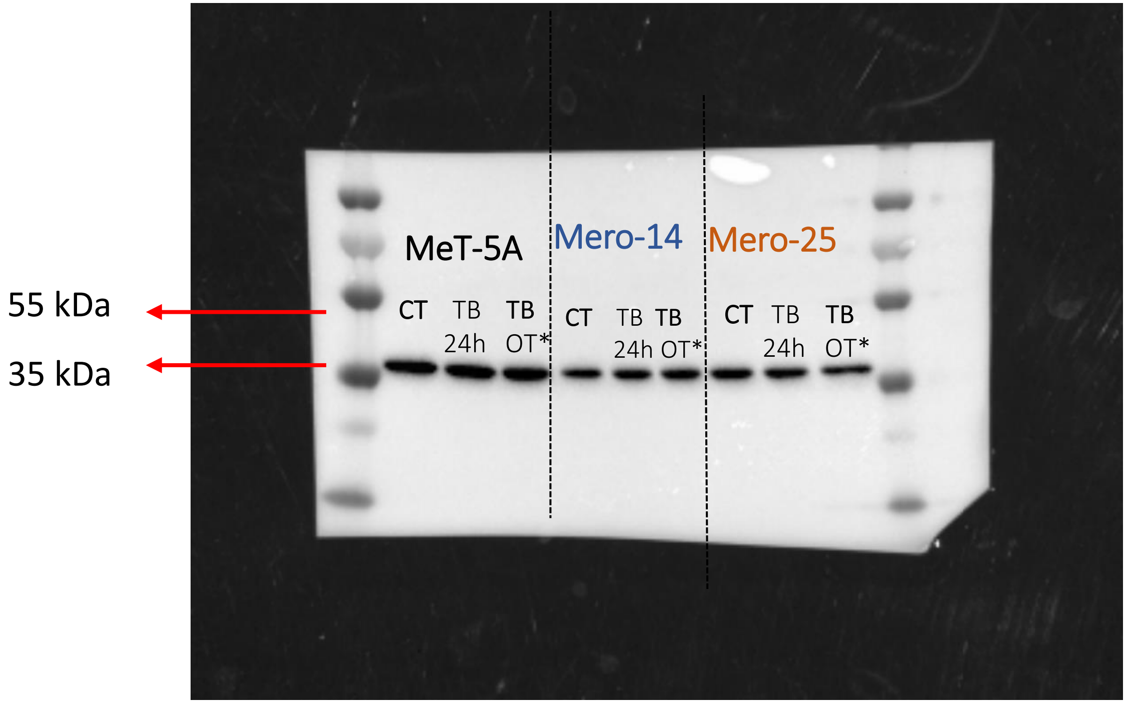

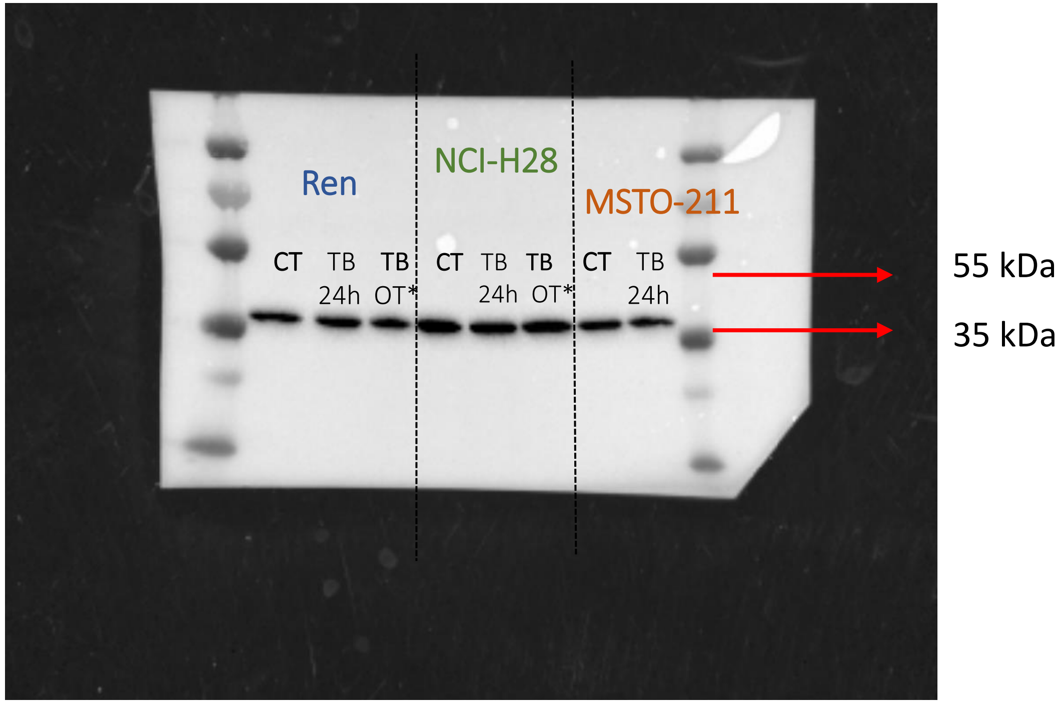

OT*: Other time point, not of interest
