# Supplementary figures and images for: Thonzonium bromide inhibits progression of malignant pleural mesothelioma through regulation of ERK1/2 and p38 pathways and mitochondrial uncoupling
Source: Cancer Cell Int. 2024 Jun 29;24:226. doi: 10.1186/s12935-024-03400-7 (PMC11218145; doi:10.1186/s12935-024-03400-7)

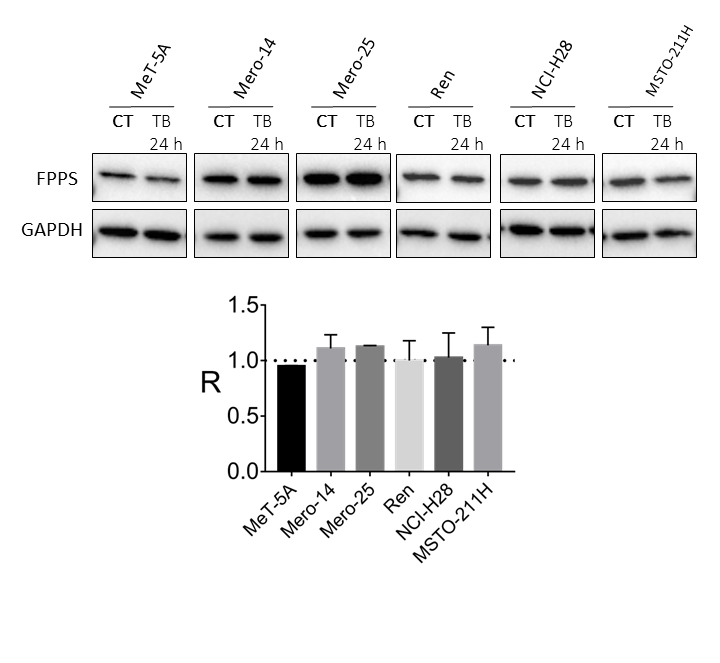

Supplement: Supplementary file 2 — Supplementary Material 2: FPPS expression in non-malignant MeT-5A and MPM cells. Western blot analysis of the expression of FPPS, a key enzyme of mevalonate pathway, after 24 hours of incubation with TB 1 μM. GAPDH was used as reference. The picture is representative of one of two experiments performed, while the histograms were generated by quantifying blots from both the experiments. The histograms report (Y-axis) the ratio (R) of the measurements obtained with Image Lab Software for FPPS relative to the controls in MeT-5A, Mero-14, Mero-25, Ren, NCI-H28 and MSTO-211H cell lines (X-axis). Controls consisted in a treatment with the vehicle only (DMSO) and are reported as dotted line. Data are expressed as mean ± standard error and the statistical significance of the comparisons between controls and treatments is indicated by asterisk (*), where =P<0.05; *=P<0.01; ***=P<0.001, compared to control treatment set to 1 (dotted line). [file 12935_2024_3400_MOESM2_ESM.tif]
